# Comparison of macrovascular invasion-free survival in early-intermediate hepatocellular carcinoma after different interventions: A propensity score-based analysis

**DOI:** 10.7150/jca.29850

**Published:** 2019-07-08

**Authors:** Yao Liu, Dongying Xue, Shanzhong Tan, Qun Zhang, Xue Yang, Yuxin Li, Bingbing Zhu, Shuaishuai Niu, Li Jiang, Xianbo Wang

**Affiliations:** 1 Center of Integrative Medicine, Beijing Ditan Hospital, Capital Medical University. Beijing 100015, China.; 2 Department of Infections Disease, Putuo Hospital, Shanghai University of Traditional Chinese Medicine. Shanghai 200062, China.; 3 Department of Integrated TCM and Western Medicine, the Second Hospital of Nanjing, Nanjing University of Chinese Medicine, Nanjing 210003, China.; 4 Department of Gastroenterology, Dongzhimen Hospital, Beijing University of Chinese Medicine. Beijing 100700, China.; 5 Department of Surgery, Beijing Ditan Hospital, Capital Medical University. Beijing 100015, China.

**Keywords:** Macroscopic vascular invasion, Hepatocellular carcinoma, Propensity score analysis

## Abstract

**Objectives**: The purpose of this study was to compare macrovascular invasion (MVI)-free survival (MFS) at the three-year follow-up in patients with hepatocellular carcinoma (HCC) who underwent hepatic resection (HR), transcatheter arterial chemoembolization (TACE), or TACE combined with radiofrequency ablation (TACE-RFA).

**Materials and Methods**: We retrospectively analyzed the medical records of 828 patients who were diagnosed with Barcelona Clinic Liver Cancer (BCLC) stage A or stage B HCC. Of these patients, 116 underwent HR, 395 underwent TACE-RFA, 239 underwent TACE, and 78 patients received conservative treatment (control group). A validation cohort of 158 patients was included. The MFS and overall survival (OS) before and after propensity score (PS) matching were evaluated using Kaplan-Meier analysis.

**Results**: The baseline characteristics between the control and TACE groups were comparable. MFS was higher in the TACE group than in the control group at the three-year follow-up (p = 0.0091), and OS was similar in the two groups (p = 0.0549). PS matching was used to generate 68 pairs of patients in the control versus HR group and 74 pairs of patients in the control versus TACE-RFA group (1-to-1 matched). MFS was significantly higher in the HR or TACE-RFA groups than in the control group (p < 0.0001 (HR versus control) and p = 0.0001 (TACE-RFA versus control), respectively). Furthermore, for patients in the HR versus TACE-RFA versus TACE groups that were generated by PS matching, the Kaplan-Meier analysis showed that MFS and OS were higher with HR or TACE-RFA than with TACE at three years. In the study, similar results were obtained in the validation cohort.

**Conclusions**: MFS and OS were higher with HR or TACE-RFA than with TACE for HCC patients without MVI.

## Introduction

Hepatocellular carcinoma (HCC) is regarded as the fifth most common malignancy worldwide and the third cause of mortality related with cancer 1. In total, 10-40% of patients are diagnosed with macrovascular invasion (MVI) associated with the portal vein, hepatic vein and/or inferior vena cava 2-4. For patients with non-resectable HCC, MVI negatively impacts the prognosis to a large extent and leads to lower median survival (two to four months) than those with no MVI whose median survival is 10-24 months 3-5. Among the types of MVI that contribute to poor outcomes, portal vein tumor thrombus is most important 4, 6 as it can cause liver dysfunction, portal hypertension, ascites, variceal rupture, hepatic encephalopathy, and/or death.

According to the internationally recognized guidelines of HCC management published by the American Association for the Study of Liver Diseases (AASLD), liver transplantation is the first choice for patients with early HCC 7, 8. Post-transplantation overall survival (OS) at 5 years among such patients can reach approximately 70% 9-11. However, in view of donor organ shortage, high cost, as well as longer waiting times for transplantation, an alternative therapeutic procedure such as hepatic resection (HR) and radiofrequency ablation (RFA) can greatly deter the progress of HCC in clinical practice 12. Moreover, two retrospective studies demonstrated that transarterial chemoembolization (TACE) combined with RFA (TACE-RFA) has an efficacy comparable to hepatectomy for primary HCC 13, 14. Recently, many centers have reported that TACE has emerged as an alternative to HR for treating HCC patients 15-18, with one study suggesting that OS following TACE can rival the OS reported after HR 15. The use of TACE has a wider application, as opposed to the Barcelona Clinic Liver Cancer (BCLC) staging system which recommends TACE only for patients with advanced HCC 19, 20. Many studies have compared the therapeutic efficacy of these interventions on survival outcomes 21, 22. However, to our knowledge, no study has compared the effects of various treatments on MVI-free survival (MFS).

In order to compare the efficacy of HR, TACE-RFA, and TACE in regulating MFS, a retrospective analysis was carried out on HCC patients without MVI. Each patient was treated by one of these three procedures at the Beijing Ditan Hospital of Capital Medical University. To minimize the potential bias in the results due to baseline confounding factors, we also analyzed propensity-score matched pairs of patients in each group.

## Materials and Methods

### Patients and treatments

The comparative study conducted here was a retrospective analysis. The diagnosis of HCC was based on the recommendations of the AASLD, and included magnetic resonance imaging (MRI), serum alpha-fetoprotein (AFP) levels, ultrasound, angiography, and computed tomography (CT) 7. The study included 828 patients who had undergone initial treatment with HR, TACE-RFA, TACE, or conservative therapy without any locoregional therapies or surgical treatment (control) at the Beijing Ditan Hospital (Beijing, China), Capital Medical University between October 2008 and November 2015. 158 patients were enrolled in the validation cohort from Putuo Hospital (Shanghai, China), Shanghai University of Traditional Chinese Medicine, and Second Hospital of Nanjing (Nanjing, China) between December 2014 and October 2015. The following criteria were used: 1 patients with HCC of BCLC A or B stage, and Child-Pugh class A or B; 2 patients without extrahepatic metastasis; 3 patients with etiologies of hepatitis C virus (HCV), hepatitis B virus (HBV), and/or related to alcohol; 4 patients with autoimmune liver disease, hepatitis A, D or E, syphilis, acquired immune deficiency syndrome were excluded; 5 patients with incomplete data or who lacked follow-up were excluded. MVI showed portal and/or hepatic vein filling defect in contrast-enhanced imaging (CT-scan or MRI), and embolic enhancement was identical or resembled to patients with primary liver cancer 23. The study was approved by each participating centre's ethics committee. All participating patients signed informed consent.

The appropriate treatment was selected by our multidisciplinary team. The following criteria were used for HR: shortage of ascites or hypersplenism, sufficient residual liver confirmed using volumetric computed tomography (CT), and Child-Pugh class A or selected B liver function. Indications for TACE-RFA were Child-Pugh A or B liver function, absent massive ascites or severe hypersplenism, and was performed in patients who unwilling to receive HR. Indications for TACE alone were Child-Pugh A or B liver function, absent massive ascites, or with gross classification of diffuse type.

### Hepatic resection procedure

Intraoperative ultrasound was routinely performed to confirm the tumor location and to evaluate the vascular anatomy of the liver. The inflow of blood to the liver was occluded using Pringle's maneuver. The clamp crushing method was used to perform the liver resection. The resection margin exceeded 1 cm. In addition, the detection of adequate drainage was confirmed.

### Radiofrequency ablation procedure

Performed RFA within 2 weeks after TACE. Conscious analgesic sedation (intravenous administration with 0.1 g pethidine hydrochloride, 0.5 mg atropine together with 10 mg diazepam), and local anesthesia (5 ml lidocaine at a concentration of 1%) were used to carry out RFA. Simultaneous RFA procedures were conducted percutaneously under nonenhanced CT (NECT) by two of four ablation experts who had 6 to 15 years of experience. The number of overlapping ablation points depended on the diameter and number of tumors, and the experts aimed to obtain an ablative margin of at least 0.5 cm in the normal tissues around the tumor, excluding the subcapsular portion and perivascular portions. At the end of the procedure, the experts ablated the needle tract to prevent bleeding and tumor seeding.

### Transcatheter arterial chemoembolization procedure

Under local anesthesia, a catheter of 4F-5F was introduced into the abdominal aorta through the superficial femoral artery utilizing the Seldinger technique. Hepatic arterial angiography was performed under fluoroscopic guidance and the guiding catheter was directed towards the coeliac artery and superior mesenteric artery. We then identified the tumor stain, the feeding artery, as well as the vascular anatomy around the tumor. The catheter was then used to direct a microcatheter towards the feeding arteries, following which a combination of lipiodol (5-10 ml), 5-fluorouracil (50 mg), and pirarubicin (30 mg) was introduced into the tumor. Subsequently, embolization was performed using gelatin sponge particles. An additional angiogram was performed at the end of the procedure to ensure full embolization of the supplying artery.

### Follow-up

Patient MFS and OS were the endpoints of the study, which were measured in months from the time of the initial diagnosis of HCC to MVI-positive findings or death. In order to assess the technical effects, enhanced MRI or contrast-enhanced computed tomography (CECT) were performed after four weeks, and follow-ups were conducted once every three months for three years. CECT or enhanced MRI, chest CT, liver function tests, and AFP tests were performed at each visit.

During the follow-up period, patients who developed recurrences, including extrahepatic recurrence, local lesion recurrence, and intrahepatic distant recurrence, would undergo specific treatments such as RFA, tumor resection, TACE, or sorafenib administration as well as conservative treatment according to the features of the recurrent tumor, liver function status, and individual patient requirements. MVI incidence referred to the interval from the time of detection of the MVI following primary treatment to the time of death or the last follow-up date.

### Statistical analysis

Continuous variables were represented by the mean ± SD, noncontinuous variables by the median value and range, and categorical variables by number and percentage. As the patients were not randomized to undergo HR, TACE-RFA, or TACE, the three treatment groups may have had confounding differences at baseline. Hence, we used logistic regression to generate propensity scores (PS) for all patients in order to reduce bias in our analyses. The three treatment groups were matched with the control group according to the generated propensity scores using a caliper width of 0.15 24. On the completion of matching, the baseline covariates were compared using the paired t-test or Mann-Whitney U test for continuous variables and the chi-square test for categorical variables. The Kaplan-Meier method was used to construct the MVI incidence curve which was compared using log-rank test. A two-tailed p value < 0.05 was considered to be statistically significant. The above statistical analyses were conducted using SPSS for Windows 22.

## Results

### Data of characteristics before and after propensity score matching

**Figure 1:**
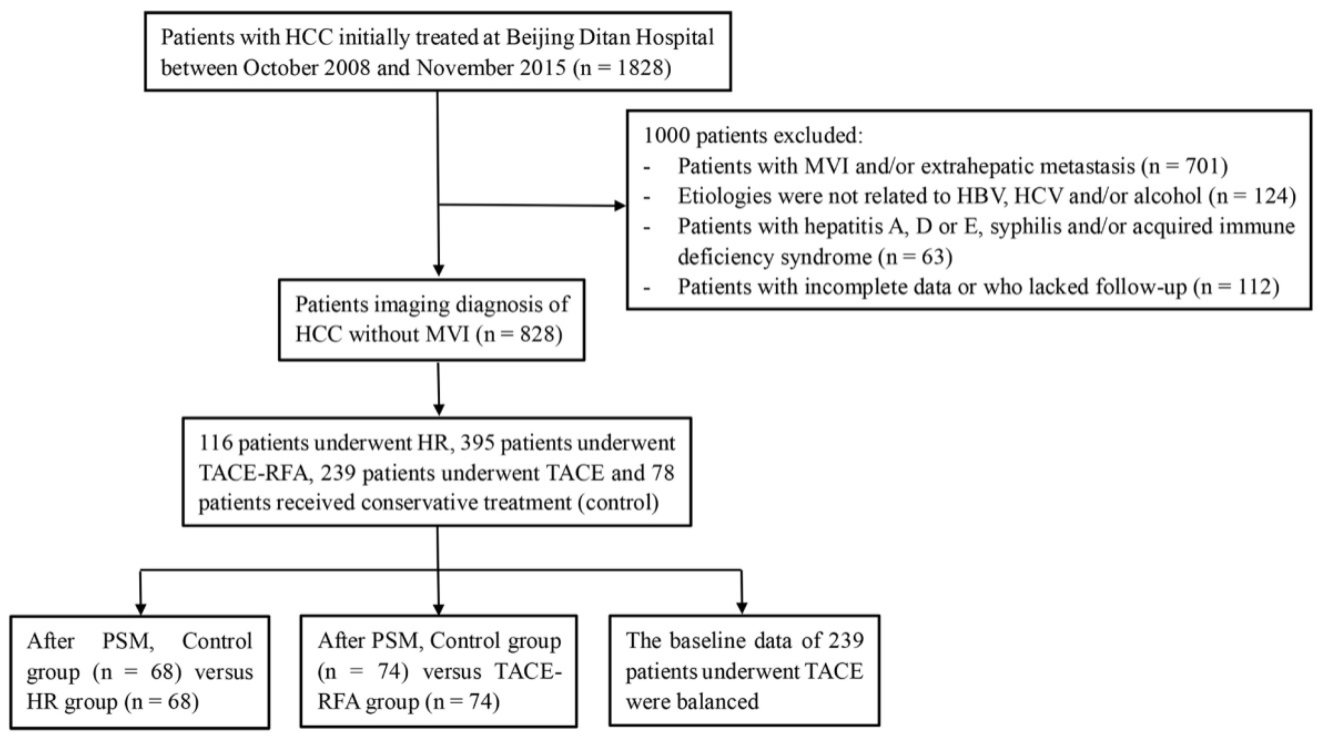
Flowchart of the treatments included in the study.

A total of 1828 patients diagnosed with HCC who underwent initial treatment at the Beijing Ditan Hospital (Beijing, China), Capital Medical University between October 2008 and November 2015 were initially investigated. Of these, 701 patients had evidence of MVI, Child-Pugh class C, and/or extrahepatic metastasis. We excluded 299 patients who did not meet the abovementioned five criteria. Finally, 828 patients were included in the study, of which 116 patients underwent HR, 395 patients underwent TACE-RFA, 239 underwent TACE, and 78 patients received conservative treatment only (did not receive any locoregional therapies or surgical treatment) (Figure 1).

The HR, and TACE-RFA groups showed obvious differences compared to the control group with respect to baseline characteristics prior to PS matching. The HR group had lower gamma-glutamyl transferase (GGT) and total bilirubin (TBIL) levels than the control group (p = 0.039 and p = 0.021) and younger age than the control group (p < 0.001). A significant difference was seen in the Model for End-Stage Liver Disease (MELD) score between the HR and control groups (p = 0.005). TBIL level, Child-Pugh class, and prothrombin time (PT) were also significantly different between the TACE-RFA and control groups. In the TACE versus control group, variables were balanced at baseline (Table 1).

In the HR versus control group matched 1:1, the propensity score model included the variables of age, GGT and TBIL levels, and MELD score. In the TACE-RFA versus control group matched 1:1, variables in the propensity score model included TBIL level, Child-Pugh class, and PT (Table 2). After PS matching, the important related characteristics became well balanced.

### Analysis of the MFS and OS

**Figure 2:**
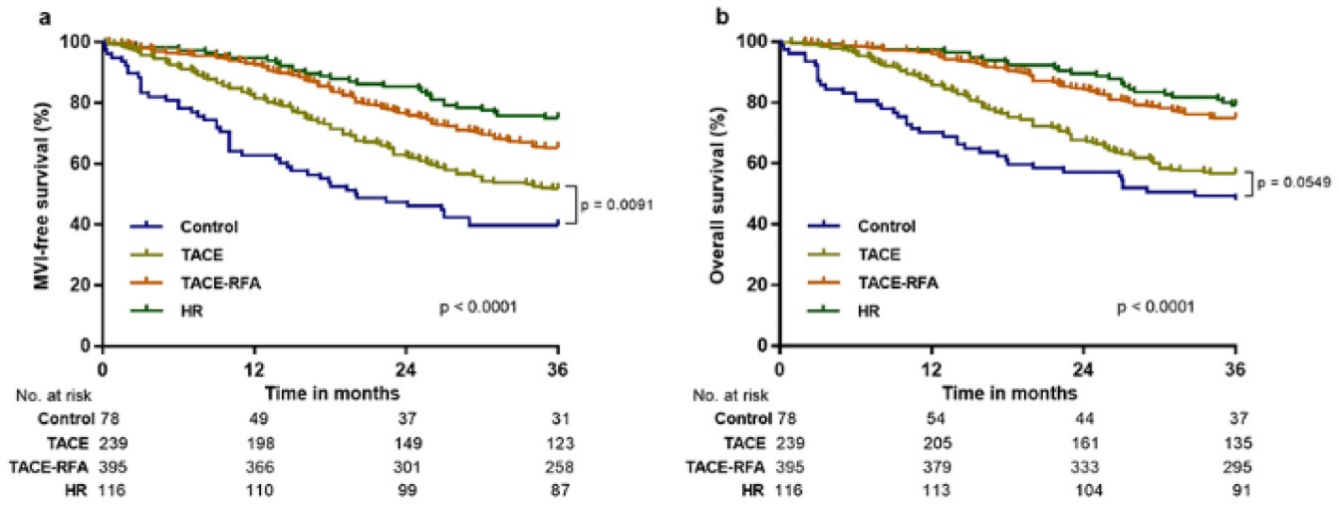
Macrovascular invasion-free survival (MFS) and overall survival (OS) in patients with hepatocellular carcinoma following various methods of treatment before propensity score (PS) matching analysis. The MFS and OS associated with hepatic resection (HR), transcatheter arterial chemoembolization (TACE) with radiofrequency ablation (TACE-RFA), TACE, and control treatment at 36 months were 75.0% and 78.4%, 65.3% and 74.7%, 51.5% and 56.5%, 39.7% and 47.4%, respectively.

During the three-year follow-up period, 87 out of 116 (75%) patients in the HR group, 258 out of 395 (65.3%) patients in the TACE-RFA group, 123 out of 239 (51.5%) patients in the TACE group, and 31 out of 78 (39.7%) patients in the control group showed MFS (Figure 2a), and 91 out of 116 (78.4%) patients in the HR group, 295 out of 395 (74.7%) patients in the TACE-RFA group, 135 out of 239 (56.5%) patients in the TACE group, and 37 out of 78 (47.4%) patients in the control group survived (Figure 2b). Before PS matching, Kaplan-Meier analysis showed that the HR and control groups exhibited significant difference in MFS and OS (p < 0.0001 for all; Figure 2). Furthermore, MFS and OS were significantly different between the TACE-RFA and control groups (p < 0.0001 for all; Figure 2). No significant difference was observed between the TACE and control groups for OS at the three-year follow-up (p = 0.0549; Figure 2b), and MFS was obviously higher in the TACE group (p = 0.0091, Figure 2a).

**Figure 3:**
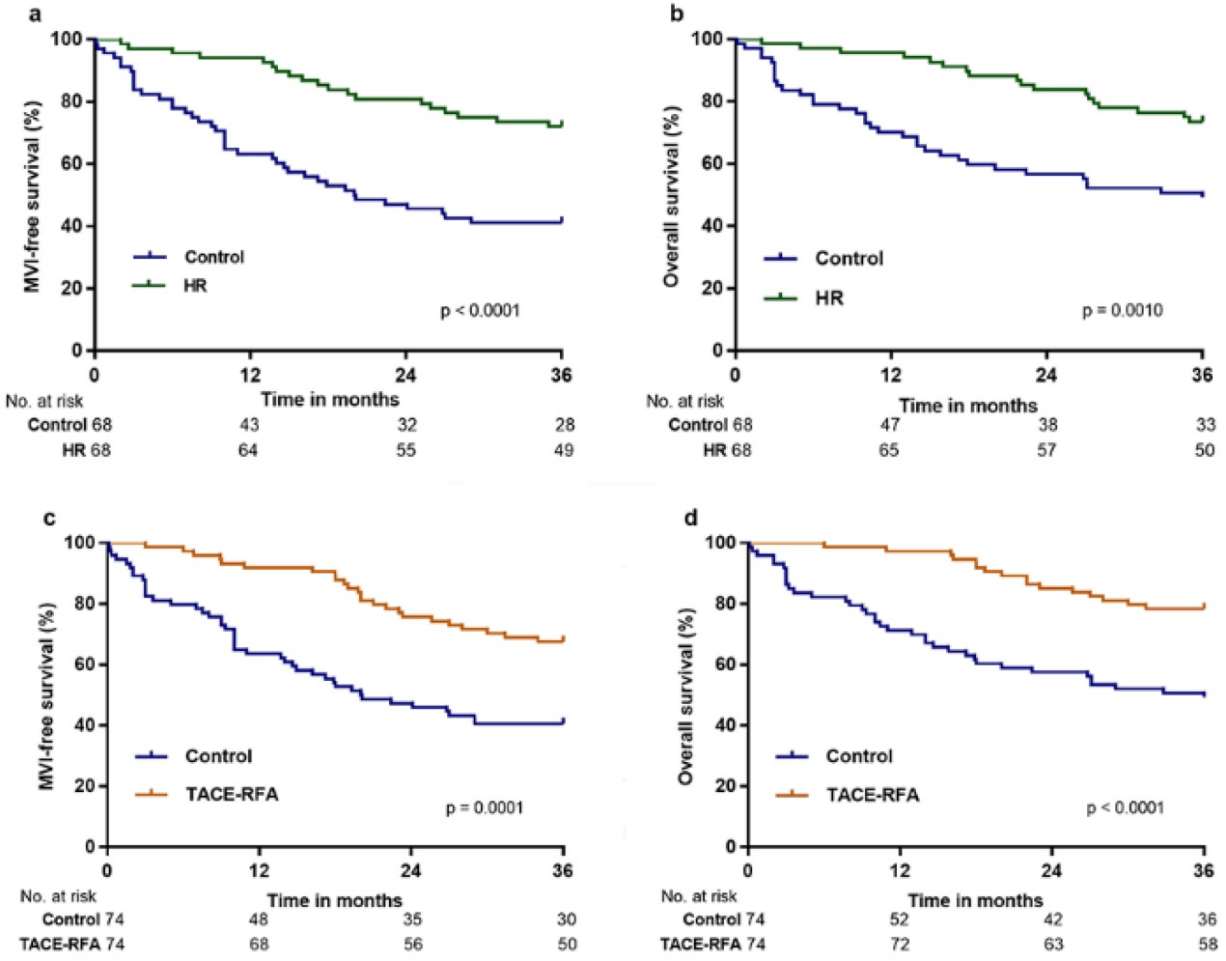
MFS and OS of patients in the HR, TACE-RFA, and TACE groups compared with those in the control group after PS matching analysis: a, MFS associated with HR vs. control; b, OS associated with HR vs. control; c, MFS associated with TACE-RFA vs. control; d, OS associated with TACE-RFA vs. control.

PS matching was employed to generate 68 pairs of patients in the control versus HR group (1-to-1 matched), for which MFS and OS were significantly higher in the HR group than in control group after three years (p < 0.0001 and p = 0.0010, respectively; Figure 3a and 3b). MFS and OS were also significantly higher in the TACE-RFA group than in the control group after three years (p = 0.0001 and p < 0.0001, respectively; Figure 3c and 3d) in the 74 pairs of patients in the control versus TACE-RFA group (1-to-1 matched).

**Figure 4:**
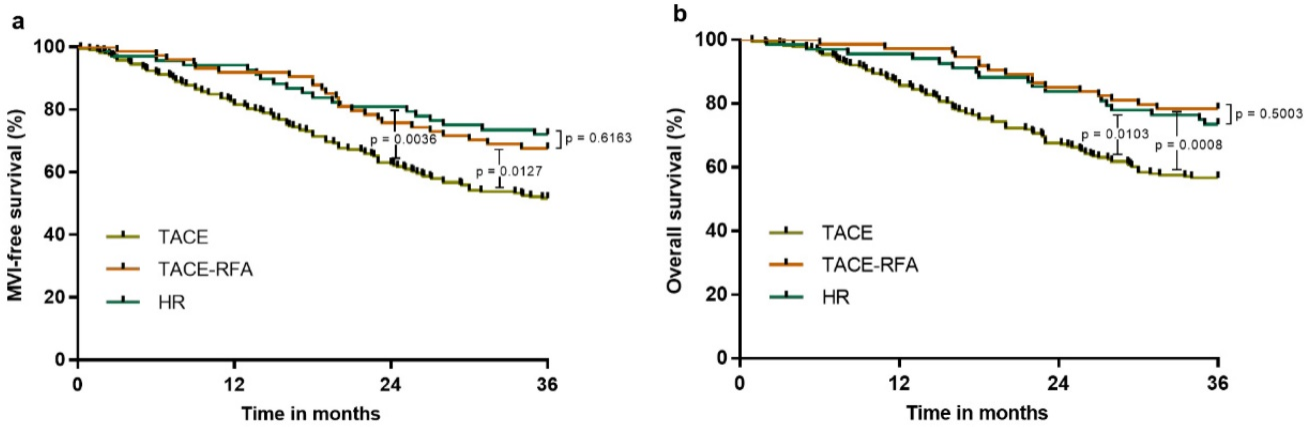
MFS and OS of patients in the HR vs. TACE-RFA vs. TACE groups after PS matching: a, MFS associated with HR vs. TACE-RFA vs. TACE; b, OS associated with HR vs. TACE-RFA vs. TACE.

Furthermore, we analyzed the three-year MFS and OS to compare the effects of the treatments of HR, TACE-RFA and TACE. The baseline characteristics for the three treatment groups were similar. The results confirmed no significant difference between the HR group and TACE-RFA group with respect to MFS and OS (p = 0.6163 and p = 0.5003, respectively; Figure 4). However, the HR and TACE-RFA groups had significantly higher MFS and OS than the TACE group (Figure 4).

### Subgroup analysis

**Figure 5:**
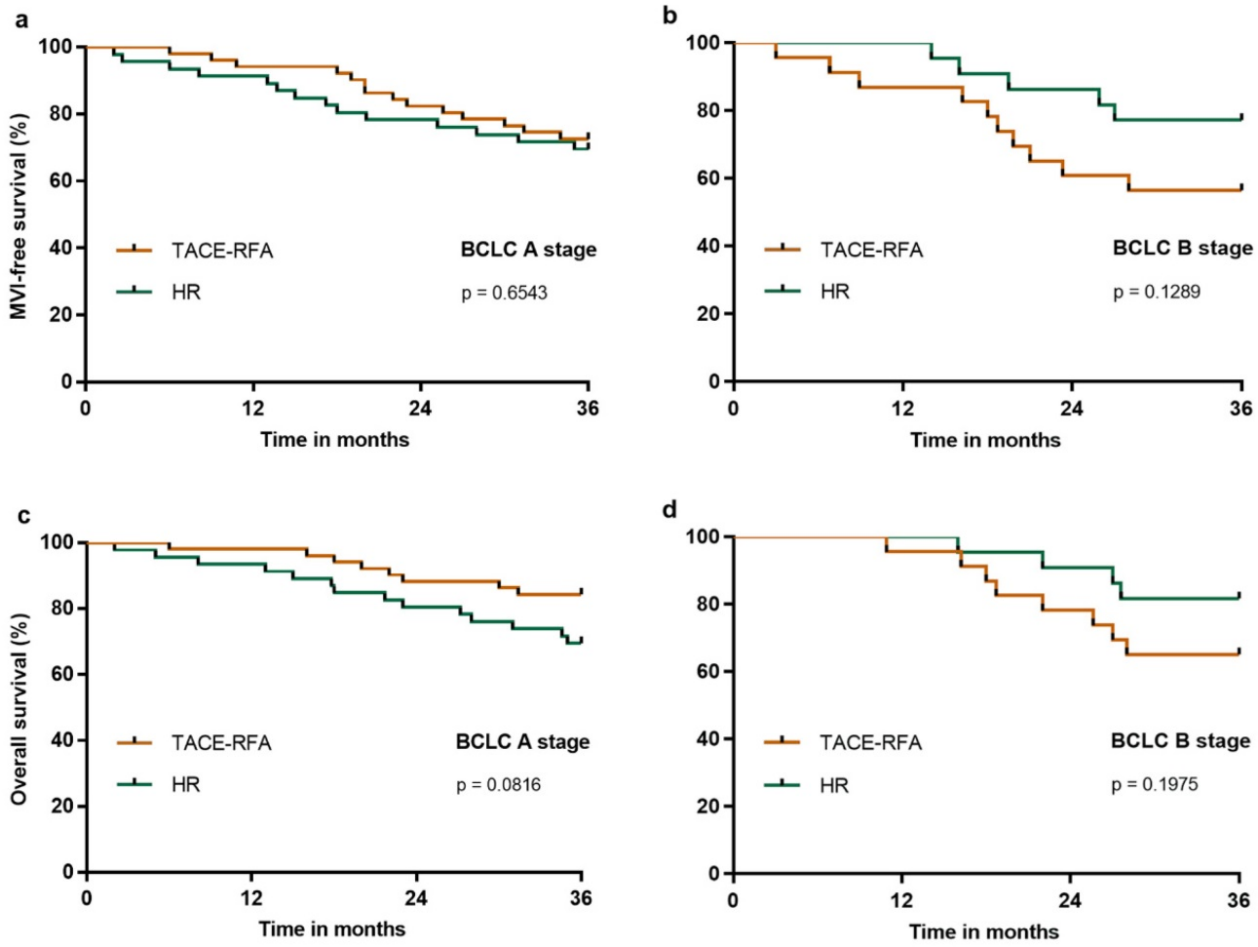
MFS and OS of patients in the HR group compared with the TACE-RFA group after PS matching analysis in BCLC A or B stages: a, MFS in BCLC A stage; b, MFS in BCLC B stage; c, OS in BCLC A stage; b, OS in BCLC B stage.

We further analyzed the three-year MFS and OS in HCC patients with BCLC A or B stage who underwent HR or TACE-RFA. Our results demonstrated that the three-year MFS was similar in the BCLC A (Figure 5a) or B (Figure 5b) stages after HR or TACE-RFA (p = 0.6543 and p = 0.1289, respectively), and that the three-year OS was also similar in the BCLC A (Figure 5c) or B (Figure 5d) stages after HR or TACE-RFA (p = 0.0816 and p = 0.1975, respectively).

### External validation cohort

**Figure 6:**
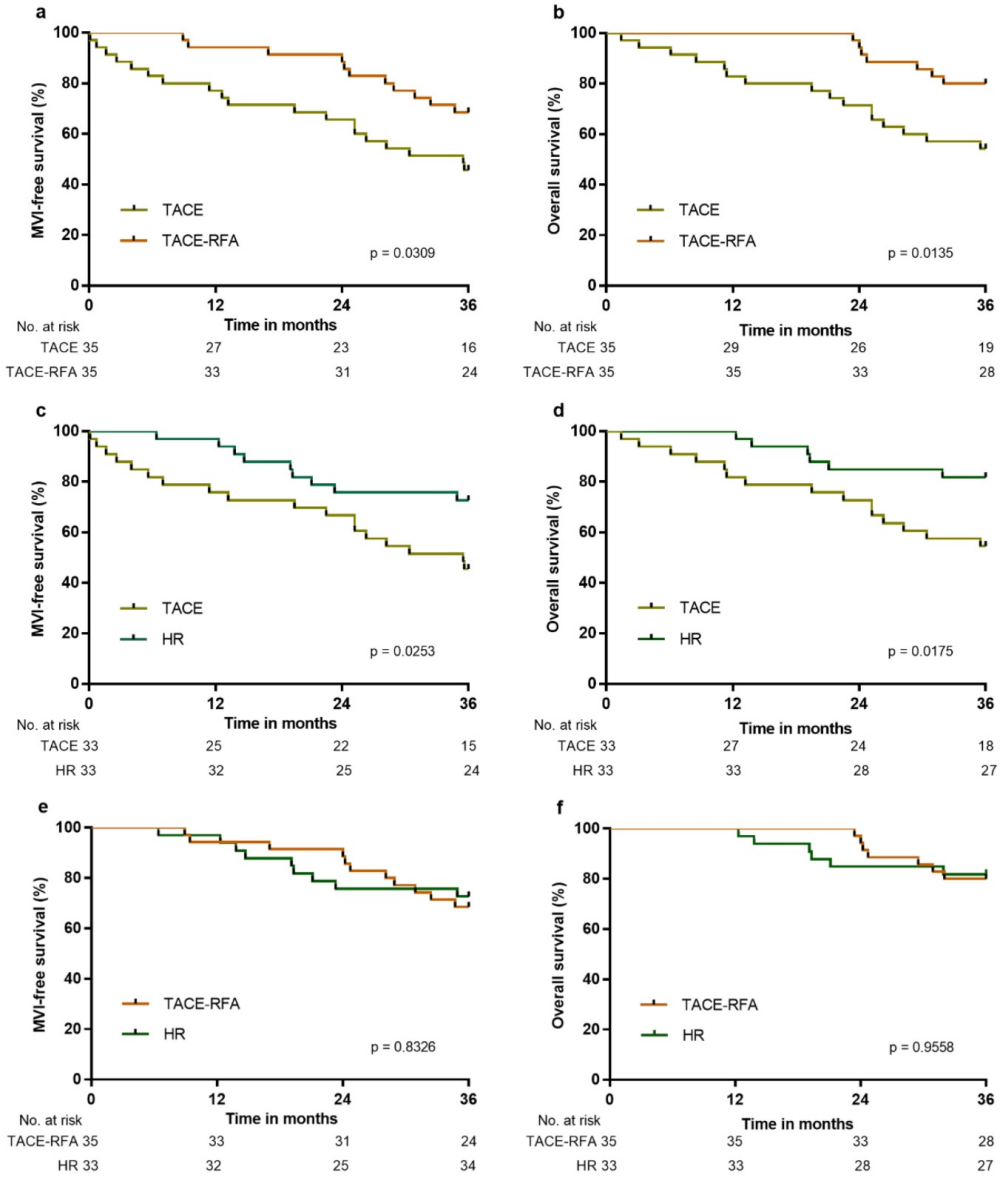
MFS and OS of patients in the external validation cohort: a, MFS associated with TACE-RFA vs. TACE; b, OS associated with TACE-RFA vs. TACE; c, MFS associated with HR vs. TACE; d, OS associated with HR vs. TACE; e, MFS associated with HR vs. TACE-RFA; f, OS associated with HR vs. TACE-RFA.

158 patients were included in the external validation cohort, of which 33 patients underwent HR, 35 patients underwent TACE-RFA, 48 underwent TACE, and 42 patients received conservative treatment. After PS matching, MFS and OS were significantly higher in the HR, TACE-RFA or TACE groups than in the control group after three years (p < 0.0001 for all), and MFS and OS were significantly higher in the HR or TACE-RFA groups than in the TACE group (Figure 6a-6d). There was no significant difference between the HR and TACE-RFA groups with respect to MFS and OS (p = 0.8326 and p = 0.9558, respectively; Figure 6e and 6f). The above results confirmed that the effects of different treatments in the external validation cohort were similar to those in the derivation cohort.

## Discussion

MVI is a common complication of HCC at advanced stages and shows a close association with intrahepatic metastasis and recurrence after transplantation, resulting in a poor prognosis 25-27. The focus of clinical investigations is aimed at the safety and survival outcomes of RFA, TACE and HR for patients with HCC. However, the role of treatment and management of HCC on the occurrence of MVI still remains unknown.

In the present study, we retrospectively enrolled 828 patients initially treated at our hospital with HR, TACE-RFA, TACE, and conservative therapy. The 3-year MFS of HCC patients treated with HR and TACE-RFA was approximately 75% and 65.3%, respectively, which was more satisfactory than the effect of TACE (51.5%) alone or control (39.7%). Hence, HR or TACE-RFA may reduce the occurrence of MVI and indirectly lengthen the survival time of HCC patients. After using PS matching to generate patient pairs that exhibited no significant differences at in the baseline, the results confirmed that HR or TACE-RFA may similarly increase the MFS and OS, which was confirmed using the external validation cohort.

Nowadays, there are various guidelines for treating HCC. The recommended treatments for early HCC based on the Milan criteria show a relative consistency. Typically, HR can contribute to a 5-year survival rate > 50 % for patients suffering early-stage HCC who satisfy the Milan criteria; thus, it is defined by the BCLC staging system as a preferred treatment 28, 29. However, a large number of studies that compared the therapeutic efficacy of HR and RFA reported a similar efficacy regarding the survival outcomes for a single small HCC of ≤3 cm 30-33. Moreover, several studies concluded that the survival rate was higher with HR than with TACE for patients suffering BCLC stage A and B HCC 34-37. However, treatments are controversial for patients with multinodular tumors or those at BCLC C stage, even if the tumors are potentially resectable.

The study aimed to compare the effects of various treatment modalities on three-year MFS, which could significantly influence the median survival time of patients with HCC. We used patients undergoing conservative treatment as the control group, with a BCLC stage of 0-D. After PS matching with the conservative treatment group, the important factors of baseline such as liver functions and BCLC staging were well balanced; the results showed that HR and TACE-RFA can similarly increase the MFS and OS at the 3-year follow-up. The results were confirmed after PS matching with HR group versus TACE-RFA group. This may be the reason that HR or TACE-RFA contributed to a higher survival rate than TACE for HCC patients.

Our study had a few limitations. First, the etiologies of patients we enrolled were HBV, HCV, and/or alcohol-related; we did not account for the possible differences in the prevalence for each treatment part or the differences in virus activity, history of antiretroviral therapy, or alcohol level intake. Second, we did not take into account the possible differences between TACE patients based on the number of treatment cycles they underwent. A previous study suggested that the OS was significantly higher for patients receiving ≥3 cycles of TACE than for those receiving <3 cycles 38. Finally, the retrospective nature of our study raised the risk of confounding, despite our use of PS matching. The study results should be modified based on the results from randomized studies that better consider patient populations at different sites.

## Conclusions

Our study confirms that the higher MFS and OS obtained using HR or TACE-RFA rather than TACE for HCC patients without MVI may help guide treatment decisions.

## Figures and Tables

**Table 1:** Baseline characteristics of patients before matching

	Control	HR			TACE-RFA			TACE	
**Variables**	**n = 78**	**n = 116**	***p* Value**		**n = 395**	***p* Value**		**n = 239**	***p* Value**
**Median Age (range)**	58(36-78)	52 (29-74)	< 0.001 ^a)^		56 (28-81)	0.341 ^a)^		57(33-84)	0.677 ^a)^
**Sex (M/F)**	64/14	96/20	0.899 ^b)^		309/86	0.450 ^b)^		195/61	0.276 ^b)^
**HBV related (yes/no)**	68/10	104/12	0.594^ b)^		331/64	0.452^ b)^		214/42	0.444^ b)^
**GGT (IU/L)**	52.1 (27.3-117.4)	36.8 (22.3-80.1)	0.039 ^c)^		46.8 (26.6-88.9)	0.374 ^c)^		46.8 (26.4-87.3)	0.350 ^c)^
**TBIL (μmol/L)**	19.3 (13.9-34.6)	15.7 (11.2-24.4)	0.021 ^c)^		16.4 (11.5-24.3)	0.011 ^c)^		17.8 (12.6-27.2)	0.183 ^c)^
**Child-Pugh class (A/B)**	47/31	83/33	0.101 ^b)^		294/101	0.011 ^b)^		158/81	0.348 ^b)^
**PT (s) (mean±SD)**	13.8 ± 2.6	13.4 ± 2.3	0.238 ^a)^		13.1 ± 1.9	0.022 ^a)^		13.3 ± 2.2	0.086 ^a)^
**MELD Score (mean±SD)**	6.3 ± 5.3	4.3 ± 3.5	0.005 ^a)^		5.2 ± 4.1	0.093 ^a)^		5.8 ± 4.7	0.431 ^a)^
**AFP (ng/mL) (< 400/≥ 400)**	60/18	99/17	0.135 ^b)^		337/58	0.065 ^b)^		195/44	0.367 ^b)^
**Tumor number (< 3/≥ 3)**	59/19	90/26	0.753 ^b)^		306/89	0.725 ^b)^		170/69	0.440 ^b)^
**Largest tumor diameter (< 5 cm /≥ 5 cm)**	65/13	96/20	0.917 ^b)^		315/80	0.466 ^b)^		179/60	0.124 ^b)^
**BCLC (A/B)**	51/27	79/37	0.693 ^b)^		279/116	0.356 ^b)^		144/95	0.418 ^b)^

Data are presented as mean ± standard deviation (SD), or median (interquartile range). ^a)^ t test. ^b)^ Chi-square test or Fisher's exact test. ^c)^ Mann-Whitney U test. HBV, hepatitis B virus; GGT, gamma-glutamyl transferase; TBIL, total bilirubin; NLR neutrophil-lymphocyte ratio; PT, prothrombin time; MELD, Model for End-Stage Liver Disease Score; AFP, α-fetoprotein; BCLC, Barcelona Clinic for Liver Cancer.

**Table 2:** Characteristics of patients after matching

	Control	HR			Control	TACE-RFA	
**Variables**	**n = 68**	**n = 68**	***P* Value**		**n =74**	**n = 74**	***p* Value**
**Median Age (range)**	57 (36-78)	55 (29-71)	0.168 ^a)^		58 (36-78)	56 (28-81)	0.367 ^a)^
**Sex (M/F)**	56/12	55/13	0.825 ^b)^		61/13	59/15	0.675 ^b)^
**HBV related (yes/no)**	60/8	61/7	0.784^ b)^		64/10	63/11	0.814^ b)^
**GGT (IU/L)**	48.0 (24.2-84.0)	34.1 (22.7-88.3)	0.354 ^c)^		52.1 (28.7-132.0)	46.0 (22.3-90.1)	0.296 ^c)^
**TBIL (μmol/L)**	18.9 (13.1-28.0)	15.7 (10.9-22.6)	0.136 ^c)^		19.7 (14.0-35.3)	18.5 (12.2-28.9)	0.255 ^c)^
**Child-Pugh class (A/B)**	45/23	46/22	0.855^ b)^		44/30	54/20	0.082 ^b)^
**PT (s) (mean±SD)**	13.0 (11.7-14.3)	12.8 (11.7-13.9)	0.462 ^c)^		13.4 (12.4-15.1)	13.0 (12.1-14.7)	0.611 ^c)^
**MELD Score (mean±SD)**	5.5 ± 4.5	5.0 ± 3.2	0.436^ a)^		6.4 ± 5.4	5.7 ± 4.6	0.412 ^a)^
**AFP (ng/mL) (< 400/≥ 400)**	54/12	59/9	0.431 ^b)^		57/17	63/12	0.282 ^b)^
**Tumor number (< 3/≥ 3)**	49/19	52/16	0.556 ^b)^		55/19	58/16	0.562 ^b)^
**Largest tumor diameter (< 5 cm /≥ 5 cm)**	56/12	56/12	1.000 ^b)^		61/13	58/16	0.534 ^b)^
**BCLC (A/B)**	44/24	46/22	0.717 ^b)^		47/27	51/23	0.487 ^b)^
